# Laparoscopic Surgical Staging of Stage I Primary Squamous Cell Carcinoma of the Vagina

**DOI:** 10.1155/DTE.1.117

**Published:** 1994

**Authors:** Joel M. Childers, Peter R. Brzechffa, Earl A. Surwit

**Affiliations:** 1 Division of Gynecologic Oncology Department of Obstetrics and Gynecology University of Arizona Tucson AZ 85724 USA; 2 2625 North Craycroft Suite 201 Tucson AZ 85712 USA

## Abstract

Vaginal carcinoma is an uncommon malignancy and one of the few gynecologic malignancies that is still clinically staged. Clinical staging, which can be difficult in some instances, is potentially inaccurate, as it has been shown to be in early endometrial and ovarian carcinoma. In addition, clinical staging can result in over- or undertreatment of the disease. The lack of standardization of treatment further compounds the issue, particularly for patients with small-volume disease. We report three patients with grade 2 or 3 small-volume primary squamous cell carcinoma of the vagina who underwent pelvic lymph node sampling for staging purposes. Each patient had lesions small enough to be considered for brachytherapy only. An average of 12 lymph nodes were removed with an average operative time of 72 minutes. All procedures were performed on an outpatient basis, and there were no intraoperative or postoperative complications. In one patient, teletherapy was added to the brachytherapy because a microscopic focus of squamous cell carcinoma was discovered in an obturator lymph node. Our initial experience indicates that laparoscopic sampling of lymph nodes in patients with early vaginal carcinoma may be helpful in preventing undertreatment of these women. Individualization of treatment can be accomplished quickly and safely on an outpatient basis, and initiation of treatment is not delayed. We believe further evaluation of laparoscopic staging of primary vaginal carcinoma is indicated.


*Diagnostic and Therapeutic Endoscopy*, Vol. 1, pp. 117–119
Reprints available directly from the publisher
Photocopying permitted by license only

© 1994 Harwood Academic Publishers GmbH
Printed in Malaysia

### Laparoscopic Surgical Staging of Stage I Primary Squamous Cell Carcinoma of the Vagina

JOEL M. CHILDERS, PETER R. BRZECHFFA and EARL A. SURWIT

*Division of Gynecologic Oncology, Department of Obstetrics and Gynecology, University of Arizona, Tucson, AZ 85724, USA*

*(Received December 27, 1993; in final form February 21, 1994)*

Vaginal carcinoma is an uncommon malignancy and one of the few gynecologic malignancies that is still clinically staged. Clinical staging, which can be difficult in some instances, is potentially inaccurate, as it has been shown to be in early endometrial and ovarian carcinoma. In addition, clinical staging can result in over- or undertreatment of the disease. The lack of standardization of treatment further compounds the issue, particularly for patients with small-volume disease. We report three patients with grade 2 or 3 small-volume primary squamous cell carcinoma of the vagina who underwent pelvic lymph node sampling for staging purposes. Each patient had lesions small enough to be considered for brachytherapy only. An average of 12 lymph nodes were removed with an average operative time of 72 minutes. All procedures were performed on an outpatient basis, and there were no intraoperative or postoperative complications. In one patient, teletherapy was added to the brachytherapy because a microscopic focus of squamous cell carcinoma was discovered in an obturator lymph node. Our initial experience indicates that laparoscopic sampling of lymph nodes in patients with early vaginal carcinoma may be helpful in preventing undertreatment of these women. Individualization of treatment can be accomplished quickly and safely on an outpatient basis, and initiation of treatment is not delayed. We believe further evaluation of laparoscopic staging of primary vaginal carcinoma is indicated.

**KEY WORDS:** laparoscopy, pelvic lymphadenectomy, vaginal carcinoma

### INTRODUCTION

Primary carcinoma of the vagina is an uncommon gynecologic malignancy. The small number of patients seen at any one institution has made standardization of treatment difficult. Currently, patients with early disease are treated either with surgery or brachytherapy alone or with a combination of brachytherapy and teletherapy (Ball and Berman, 1982; Perez *et al.*, 1973; Marcus *et al.*, 1978). The need for external radiation to treat nodal metastases remains unclear.

Most gynecologic malignancies are now surgically staged. Analysis of treatment results is believed to be more accurate using surgical staging. In addition, individualization of treatment is based on a more accurate assessment of disease status. Surgical staging of vaginal carcinoma has not been reported despite existing controversy on the ideal management of patients with early disease.

Operative laparoscopy recently has been used to stage patients with other gynecologic malignancies (Childers *et al.*, 1992); Childers, Brzechffa *et al.*, 1993; Querleu, 1993; Childers, Tran *et al.*, 1993). Initial reports indicate that this minimally invasive surgical approach is safe, adequate, and may be advantageous for the patient.

We report the results of three patients with stage I primary squamous cell carcinoma of the vagina who were surgically staged by laparoscopic pelvic lymph node sampling. All patients were considered candidates for brachytherapy only.

### MATERIALS AND METHODS

Between July 1992 and July 1993, three patients with stage I squamous cell carcinoma of the vagina were considered candidates for laparoscopic pelvic lymph node sampling. All three patients had small lesions (2–3 cm) located in the upper vagina. Two patients had anterior wall lesions, and one had a left-sided lesion. The tumors were poorly differentiated in two patients and moderately well differentiated in the third (See Table 1).

Address for correspondence: Dr. Joel M. Childers 2625 North Craycroft, Suite 201, Tucson, AZ 85712.

**Table 1**

| Patient | Age (years) | Weight (pounds) | Tumor size(cm) | Tumor location | Tumor grade | Nodes Number | Nodes Status | Hospital stay (days) | Operative time (minutes) |
|---|---|---|---|---|---|---|---|---|---|
| 1 | 53 | 162 | 2×2 | Anterior upper 1/3 | 3 | 10 | One microscopic positive | 0 | 45 |
| 2 | 47 | 145 | 2×2 | Anterior upper 1/3 | 2 | 7 | Negative | 0 | 105 |
| 3 | 75 | 134 | 2×3 | Left wall upper 1/3 | 3 | 18 | Negative | 0 | 65 |

The patients were 47, 53, and 75 years of age; none was obese, and all were in good medical health. One patient (no. 2) had a previous hysterectomy, which was performed for benign reasons. Both younger patients were smokers.

The patients were counseled regarding management options, including brachytherapy alone, combined brachytherapy and teletherapy, or radiation therapy based on the presence or absence of metastatic disease in their pelvic lymph nodes removed via laparoscopy.

Each patient received a mechanical bowel preparation consisting of two days of clear liquids and 240 mL of magnesium citrate each day for two days before the procedure. All were admitted on the morning of surgery, and all procedures were performed with the patient in the supine position under general anesthesia. No prophylactic antibiotics were administered. Prior to lymphadenectomy, the two patients with anterior vaginal wall lesions underwent urethroscopy and cystoscopy.

The four-trocar technique, as previously described, was used in all cases (Childers *et al.*, 1992). The round ligament was not transected in the two patients with uteri in situ. Pelvic washings were obtained for cytology before the lymphadenectomy was performed. A bilateral transperitoneal pelvic lymph node sampling was performed in all three patients, as previous described (Childers *et al.*, 1992). No peritoneal closures were performed, and no retroperitoneal drains were placed.

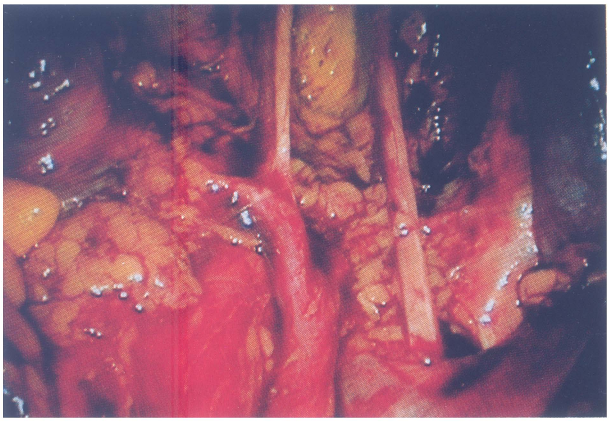

**Figure 1** The right retroperitoneal space after a transperitoneal laparoscopic pelvic lymphadenectomy. The hypogastric superior vesicle and uterine artery can clearly be seen. The obturator nerve is seen exiting behind the internal and external iliac veins and disappearing into the obturator fossa.

### RESULTS

Laparoscopic staging was successfully accomplished in all three patients. Estimated blood loss was 50 mL for each procedure, and there were no intraoperative, early, or late postoperative complications. None of the lymphatic tissue removed was grossly suspicious for metastatic carcinoma. The lymph node count ranged from 7 to 18, with an average of 11.7. Microscopically, all nodes were negative except one left obturator node in patient no. 1, which contained microscopic squamous cell carcinoma. Operative times ranged from 45 to 105 minutes, with an average of 72. All patients were discharged on the day of surgery. Each patient subsequently underwent intracavitary radiotherapy. External-beam radiotherapy was added for patient no. 1 because of the obturator lymph node that contained metastatic disease. Thus far, no patient has had recurrence of disease, and patients no. 1 and 2 are more than one year postsurgery.

### DISCUSSION

The treatment of primary vaginal carcinoma, a relatively rare neoplasm of the female genital tract, has not been standardized (Perez *et al.*, 1982). The disease Accounts for only 1–2% of all gynecologic cancers, making it difficult to study patients prospectively. The rarity of this malignancy also requires retrospective analysis to span several decades. The changes in radiation equipment and techniques over this time make interpretation of data at a single institution and comparison of results from various institutions difficult.

Patients with stage I disease traditionally have done well. Currently, five-year survivals of 70% to 100% are being reported (Nori *et al.*, 1983; Prempree, 1982;

Puthawala *et al.*, 1983; Benedet *et al.*, 1983; Gallup *et al.*, 1987; Pride *et al.*, 1979; Rubin *et al.*, 1985; Spirtos *et al.*, 1989). The tumor control and survival that these patients enjoy is often achieved with brachytherapy alone. Many authors advocate treating some patients with intracavitary and/or interstitial sources alone (Brown *et al.*, 1971; Rutledge, 1967; Perez and Camel, 1982). Perez *et al.* reported the same tumor control in the pelvis of stage I lesions with or without the addition of external-beam radiotherapy (Perez and Camel, 1982).

Even some patients with stage II disease have been treated with brachytherapy alone. Perez *et al.* advocated a modification of the International Federation of Gynecology and Obstetrics (FIGO) staging system to separate patients with stage II disease who do not need external-beam radiotherapy. The authors believe that patients with stage IIA disease (subvaginal infiltration not involving the parametrium) can be treated with brachytherapy alone, whereas patients with stage IIB disease (parametrial infiltration not involving the pelvic wall) require the addition of external-beam radiotherapy to reduce tumor size and sterilize disease in pelvic lymphatics. Pride *et al.*, using this staging modification retrospectively, reported a 65% five-year survival rate for patients with stage IA and IIA disease. This rate dropped to 31% for patients with stage IIB disease (Pride *et al.*, 1979).

Vaginal carcinoma is one of the few gynecologic malignancies that is still clinically staged. Even using the Perez-modified FIGO staging, the limitations of clinical staging are obvious. This classification system does not take into account the status of regional lymph nodes. The limitations of traditional radiologic imaging techniques in detecting metastatic lymph node involvement are well known. These limitations in clinical staging possibly could account for the wide variation reported in the literature of survival rates and stage distribution.

Currently, the management of primary squamous cell carcinoma of the vagina is individualized. Factors such as previous irradiation, stage, size, location, and tumor grade are important considerations. The addition of lymph node status would allow even further individualization of therapy. With this information, the decision to treat patients with small-volume disease with local treatment only (brachytherapy or surgery) would be based on more sound scientific information. Only patients with lymph nodes containing metastatic disease then would be treated with external-beam radiotherapy.

Lymph node sampling in patients with vaginal carcinoma could be performed in a number of ways. The transperitoneal technique we used currently is a popular method of laparoscopic lymphadenectomy. However, a retroperitoneal approach, whether or not endoscopic, could easily be employed and may offer an advantage to the patient who subsequently receives teletherapy. Inguinal nodes should be sampled in patients with distal vaginal involvement.

We believe that surgical staging may benefit patients with primary vaginal carcinoma just as it has for patients with early vulvar, endometrial, and ovarian carcinoma. Our initial experience with laparoscopic staging of stage I primary squamous cell carcinoma of the vagina indicates that the additional information obtained aids in individualization of treatment, possibly allowing for lower morbidity and an improved survival rate. Our data support further investigation into the surgical staging of primary vaginal carcinoma.

### REFERENCES

Ball H. G., Berman M. L. (1982) Management of primary vaginal carcinoma. *Gynecol Oncol*, 14:154–163.

Benedet J. L., Murfy K. J. *et al.* (1983) Primary invasive carcinoma of the vagina. *Obstet Gynecol*, 62:715–719.

Brown G. R., Fletcher G. H. *et al.* (1971) Irradiation of in situ and invasive squamous cell carcinoma of the vagina. *Cancer*, 28:1278–1283.

Childers J. M., Brzechffa P. R. *et al.* (1993) Laparoscopic assisted surgical staging (LASS) of endometrial carcinoma. *Gynecol Oncol*, 51:33–38.

Childers J. M., Hatch K. D. *et al.* (1992) The role of laparoscopic lymphadenectomy in the management of cervical carcinoma. *Gynecol Oncol*, 47:38–43.

Childers J. M., Tran, A. N. *et al.* (1993) Laparoscopic para-aortic lymphadenectomy in gynecologic malignancies. *Obstet Gynecol*, 82:741–747.

Gallup D. G., Talledo O. E. *et al.* (1987) Invasive squamous cell carcinoma of the vagina: a 14-year study. *Obstet Gynecol*, 69:782–785

Marcus R. B., Million R. R. *et al.* (1978) Carcinoma of the vagina. *Cancer*, 42:2507–2512.

Nori D., Hilaris B. S. *et al.* (1983) Radiation therapy of primary vaginal carcinoma. *Int J Radiat Oncol Biol Phys*, 9:1471–1475.

Perez C. A., Arneson A. N. *et al.* (1973) Malignant tumors of the vagina. *Cancer*, 31:36–44.

Perez C. A., Camel H. M. (1982) Long-term follow-up in radiation therapy of carcinoma of the vagina. *Cancer*, 49:1308–1315.

Perez C. A., DiSaia P. J. *et al.* (1982) Carcinoma of the vagina. In: DeVita V. T., Hellman S., Rosenberg S. A. (eds.): *Cancer Principles and Practice of Oncology*. J.B. Lippincott Co., Philadelphia.

Prempree T. (1982) Role of radiation therapy in the management of primary carcinoma of the vagina. *ACTA Radiol Oncol*, 21:195–201.

Pride G. L., Schultz A. E. *et al.* (1979) Primary invasive squamous carcinoma of the vagina. *Obstet Gynecol*, 53:218–225.

Puthawala A., Syed N. *et al.* (1983) Integrated external and interstitial radiation therapy for primary carcinoma of the vagina. *Obstet Gynecol*, 62:367–372.

Querleu D. (1993) Laparoscopic para-aortic node sampling in gynecologic oncology: a preliminary experience. *Gynecol Oncol*, 49:24–29.

Rubin S. C., Young J. *et al.* (1985) Squamous carcinoma of the vagina: treatment, complications, and long-term followup. *Gynecol Oncol*, 20:346–353.

Rutledge F. (1967) Cancer of the vagina. *Am J Obstet Gynecol*, 97:635–655.

Spirtos N., Doshi B. P. *et al.* (1989) Radiation therapy for primary squamous cell carcinoma of the vagina: Stanford University experience. *Gynecol Oncol*, 35:20–26.

